# Health-related quality of life inequalities by sexual orientation: Results from the Barcelona Health Interview Survey

**DOI:** 10.1371/journal.pone.0191334

**Published:** 2018-01-24

**Authors:** Marc Marti-Pastor, Gloria Perez, Danielle German, Angels Pont, Olatz Garin, Jordi Alonso, Mercè Gotsens, Montse Ferrer

**Affiliations:** 1 IMIM (Hospital del Mar Medical Research Institute), Health Services Research Group, Barcelona, Spain; 2 CIBER en Epidemiología y Salud Pública (CIBERESP), Madrid, Spain; 3 Universitat Autonoma de Barcelona (UAB), Barcelona, Spain; 4 Public Health Agency of Barcelona, Barcelona, Spain; 5 Universitat Pompeu Fabra, Barcelona, Spain; 6 Department of Health, Behavior and Society, Johns Hopkins Bloomberg School of Public Health, Baltimore, Maryland, United States of America; 7 Institute of Biomedical Research (IIB Sant Pau), Barcelona, Spain; Scientific Institute of Public Health (WIV-ISP), BELGIUM

## Abstract

**Background:**

Studies on health-related quality of life (HRQoL) inequalities according to sexual orientation are scarce. The aim of this study was to assess HRQoL inequalities between lesbian, gay, and bisexual (LGB) people and heterosexuals in the 2011 Barcelona population, to describe the extent to which sociodemographic characteristics, health-related behaviors, and chronic conditions could explain such inequalities, and to understand if they are sexual orientation inequities.

**Methods:**

In the 2011 Barcelona Health Interview Survey 3277 adults answered the EQ-5D, which measures five dimensions of HRQoL summarized into a single utility index (1 = perfect health, 0 = death). To assess HRQoL differences by sexual orientation we constructed Tobit models for the EQ-5D index, and Poisson regression models for the EQ-5D dimensions. In both cases, nested models were constructed to assess the mediator role of selected variables.

**Results:**

After adjusting by socio-demographic variables, the LGB group presented a significantly lower EQ-5D index than heterosexuals, and higher prevalence ratios of problems in physical EQ-5D dimensions among both genders: adjusted prevalence ratio (aPR) = 1.70 for mobility (p = 0.046) and 2.11 for usual activities (p = 0.019). Differences in mental dimensions were only observed among men: aPR = 3.15 for pain/discomfort (p = 0.003) and 2.49 for anxiety/depression (p = 0.030). All these differences by sexual orientation disappeared after adding chronic conditions and health-related behaviors in the models.

**Conclusion:**

The LGB population presented worse HRQoL than heterosexuals in the EQ-5D index and most dimensions. Chronic conditions, health-related behaviors and gender play a major role in explaining HRQoL differences by sexual orientation. These findings support the need of including sexual orientation into the global agenda of health inequities.

## Introduction

Sexual orientation is a social determinant of health inequities which influences different morbidity and mortality outcomes [1,2]. Even in western countries, where progress in social rights has been large and rapidly implemented from the end of the 20th century, the lesbian, gay, and bisexual (LGB) population presented worse health than the heterosexual one [3–6]. Health inequalities among the LGB population have been reported for mental health [4,6,7], chronic conditions [3], and health-related behaviors [7,8]; but very few studies have assessed health-related quality of life (HRQoL) [3,4,6,9].

HRQoL is a broad and multidimensional concept which describes the physical, social, and psychological aspects of well-being and functioning [10]. It is considered an ultimate and comprehensive outcome on the conceptual model of health [11]. However, only two studies on sexual orientation inequalities have considered HRQoL as a whole [3, 9], while others [4,6] just included selected HRQoL dimensions. The California Quality of Life Survey [4] only reported the physical component of the Short Form-12 Health Survey (SF-12), and the combined meta-analysis of health surveys from United Kingdom (UK) only reported anxiety/depression from the EuroQol-5 Dimensions (EQ-5D) [6]. The Dutch National Survey of General Practice assessed both the physical and the mental health components covered by the Short Form-36 Health Survey (SF-36), [3] and the United States Growing Up Today Study [9] reported the EQ-5D index. A major feature of the EQ-5D instrument lies on its single index (based on societal preference utilities), which allows the calculation of quality-adjusted life years (QALYs) [12].

It is important to distinguish between inequality and inequity in health among population groups [13]. Inequity refers to inequalities which are avoidable and unfair, since they are consequence of the different opportunities and resources that people have due to their social position [14]. The aim of our study was to assess HRQoL inequalities between LGB and heterosexuals in the 2011 Barcelona population, to describe the extent to which sociodemographic characteristics, health-related behaviors, and chronic conditions could explain such inequalities, and to understand if they are sexual orientation inequities.

Following the structural framework proposed by Mule et al. [15], we hypothesize worse HRQoL in LGB than their heterosexual counterparts. Health determinants such as age, education level, country of birth, partnership status, and social support can be potential confounders when assessing health inequalities by sexual orientation [16]. Furthermore, age [17] and gender [4,18] can modify the effect of sexual orientation in health. Despite the favorable social climate of Barcelona toward sexual minorities in the world [19], our hypothesis is that discrimination by sexual orientation may lead to increased vulnerabilities, such as distress and worse health-related behaviors, which result in higher prevalence of chronic conditions and, finally, worse HRQoL.

## Material and methods

### Study population

Data used in this study came from the Barcelona Health Interview Survey (BHIS) 2011 edition. It is a cross-sectional study periodically performed in Barcelona [20], a city in the north-east of Spain with about 1.5 million inhabitants. A representative sample of the non-institutionalized population, older than 15 years, was surveyed through computer-assisted personal interviews administered face to face by accredited interviewers in the respondent’s home.

To ensure territorial representativeness, the sample was stratified by municipal districts. A random sampling strategy was applied, using a simple extraction system from the municipal census, between January 2011 and January 2012 taking into account gender and age distribution. The sample size was estimated at 4,000 individuals (relative error margin of 1.55% with a confidence level of 95.5%). The BHIS is an official statistical activity, and data confidentiality is guaranteed by the Spanish Law Number 23/1998.

### Variables and measurement instruments

#### The EQ-5D

The EQ-5D covers five dimensions of health (mobility, self-care, usual activities, pain/discomfort, and anxiety/depression) with three levels of severity, from none to extreme problems. Its validity and reliability have been demonstrated in general population health surveys [21,22]. We used the conventional Time Trade Off preference values from the Spanish general population [23] which produced a single preference-based index ranging from 1 (best health state) to negative values, where 0 is equal to death.

#### Sexual orientation

Sexual orientation (1) was assessed from responses to the question of the National Survey of Sexual Attitudes and Lifestyles of United Kingdom [24]: *“Which of the following statements do you feel more identified with*?*”*, *with six response options considering attraction only to the opposite sex*, *usually to the opposite sex*, *equally to the same and opposite*, *usually to the same*, *only to the same sex*, *or rather not answering*. These were dichotomized into heterosexual for those responding the first option, and LGB for the other four sexual attraction combinations stated.

#### Health-related behaviors

Information about lifetime use of the following psychoactive drugs was gathered trough five groups of substances: tranquilizers, hashish or marihuana, cocaine or by-products, amphetamines or similar, and heroine.

Alcohol consumption during the past year on working days and weekends separately was collected, and weekly consumption was calculated with the formula: Standard drink units (1 unit = 10 g alcohol) * Number of drinks * frequency weekly [25]. Alcohol consumption was categorized into non-drinker, moderate, or risk drinker (>17 or >28 alcohol units/week for women and men, respectively).

Body mass index (BMI) was also considered, since the LGB population has consistently been shown to have weight differences with respect to heterosexuals [26]. BMI was divided into low/normal and overweight/obesity applying the cut-off point of 25.

#### Socio-demographic variables

Participants in 2011-BHIS were asked about their gender (women or men), age, country of birth, education level, social class, social support, and whether they were living with a partner. The maximum aggregation of categories for each variable was used to avoid cells with zero individuals because the number of LGB participants was small (n = 77).

Social class (manual and non-manual workers) was based on the Spanish National Classification of Occupations 2011 using a neo-Weberian approach [27]. Country of birth was categorized into low vs high income countries according to the GNI per capita (World Bank Atlas Method for the 2011 fiscal year) [28]. Social support was assessed with the Duke-UNC Functional Social Support Questionnaire composed of eight-items, using the recommended percentile 15 [29] as the cut-off to define low social support.

#### Chronic conditions

Participants in the 2011-BHIS were asked about 15 chronic conditions. A summary indicator based on the number of reported chronic conditions was categorized according to sample distribution into 5 groups: none, 1, 2, 3–4, and 5 or more chronic conditions.

### Statistical analysis

The 2011-BHIS sample size allows the detection of differences of 0.07 points on the EQ-5D index mean (estimated as the minimal important difference [30]) between LGB (N = 77) and heterosexual (N = 3,200) groups with alpha risk 0.05 and beta of 0.2. To restore the representativeness of the Barcelona population, a weighting factor was applied for age, gender, and municipal district. Unweighted frequencies and weighted percentages were calculated. Differences between participants with and without information on sexual orientation, and differences between LGB respondents and heterosexual counterparts were tested using χ^2^. Due to the imbalance mainly in age between LGB and heterosexual groups, the percentages of chronic conditions and health-related behaviors were adjusted by age and gender using logistic regression models.

To assess differences by sexual orientation on HRQoL, we built censored linear regression models (Tobit) with EQ-5D index, and Poisson regression models with EQ-5D dimensions. Censored linear regression models (Tobit) were used due to the right-skewed distribution of EQ-5D index (dependent variable). Marginal effects were obtained from the Tobit model as averaged individual marginal effects [31] to restore the original range of the EQ-5D index. The original three-level response scale of EQ-5D dimensions was dichotomized into “no problems” versus “moderate/extreme problems”, and it was included as the dependent variable in Poisson regression models with robust error variance. These models were used to estimate the prevalence ratio [32], which is more interpretable and easier to communicate than an odds ratio (obtained with logistic regression models) in cross-sectional studies.

In all cases, nested models were constructed to assess the mediator role of selected variables: first including only sexual orientation, which is the principal explanatory variable (Model 1), then adding sequentially gender and age (Model 2), socio-demographic variables (education level, country of birth, and married or in sentimental partnership in Model 3), number of chronic conditions (Model 4), and health-related behaviors (smoking status, alcohol consumption, and psychoactive drug consumption in Model 5). These nested models were compared with the immediately previous one using the log-likelihood ratio test. Interactions of sexual orientation with gender and age were tested.

Finally, a sensitivity analysis was performed by excluding participants that reported being attracted to the same sex only sometimes, to test validity of sexual orientation definition (S1 File). A sensitivity analysis was also carried out for comparison purposes by matching LGB and heterosexual individuals with propensity score. The primary objective of this analysis was to maximize the balance in the distribution of possible confounders between LGB and heterosexual groups. A logistic regression model was constructed to estimate the conditional probability of belonging to each group (propensity score) by including socio-demographic characteristics as independent variables. Quartiles of this conditional probability were used to define four propensity score categories. LGB individuals were matched with heterosexual counterparts (ratio 1:5) according to propensity score quartile, age group, and gender. The five heterosexual participants for each LGB individual were randomly selected, giving priority to individuals with less potential pairs. In this sensitivity analysis, conditional logistic regression was used to estimate odds ratio of reporting EQ-5D problems to take into account the matching, instead of the Poisson regression with robust error variance used in the main analysis strategy (S2 File).

## Results

Of the 3,524 participants, 247 (7%) did not state their sexual orientation. The non-respondents were older (*p*<0.001), with a lower education level (*p* = 0.021), less frequently married or in a sentimental partnership (*p*<0.001), and with lower social support (*p* = 0.011) than participants who responded (Table 1).

**Table 1:** Socio-demographic differences between participants with and without information on sexual orientation. Unweighted frequencies and weighted percentages.

	Data on sexual orientation N = 3277	No data on sexual orientation N = 247	*p-value*
**Sex**			
Men	1559 (46.9%)	126 (49.2%)	0.461
Women	1718 (53.1%)	121 (50.8%)	
**Age group**			
*15–34 years*	929 (29.0%)	56 (25.7%)	<0.001**
*35–64 years*	1597 (48.3%)	95 (37.2%)	
*65 years and over*	751 (22.7%)	96 (37.2%)	
**Education level**			
*Primary or less*	1346 (42.2%)	120 (51.2%)	0.021*
*Secondary*	833 (25.6%)	47 (21.0%)	
*University or more*	1080 (32.3%)	69 (27.8%)	
**Social class**			
*Non manual*	1708 (56.1%)	120 (56.7%)	0.870
*Manual*	1325 (43.9%)	92 (43.3%)	
**Married or in sentimental partnership**			
Yes	1904 (57.2%)	104 (41.2%)	<0.001**
No	1373 (42.8%)	143 (58.8%)	
**Country of birth**			
High income countries	2820 (87.1%)	222 (90.0%)	0.189
Low income countries	445 (12.9%)	24 (10.0%)	
**Social support**			
*Social support (>P15)*	2752 (84.5%)	222 (90.4%)	0.011*
*Low social support (≤P15)*	525 (15.5%)	25 (9.6%)	

**Bold**: significant p-value (*p-value<0.05; **p-value<0.01).

Of 3,277 respondents, 3,200 only felt attracted to the opposite sex and 77 (2.3%) became attracted to the same sex with varying frequency. This latter group was composed of: 34 who only felt attracted to the same sex, 8 only sometimes to the opposite sex; 13 felt equally attracted to both sexes; and 22 only sometimes to the same sex. Characteristics of the heterosexual and LGB groups are shown in Table 2. Differences by sexual orientation were not significant regarding gender and social class, but they were significant for several other variables. The LGB group was younger (56.6 vs 28.4% <35 years old; p<0.001), with higher education level (44.7% vs 32.0% university; *p* = 0.013), was less frequently married or in a sentimental partnership (25.0% vs 58.0%; *p* = 0.001), and came more frequently from low-income countries (22.1% vs 12.7%; *p* = 0.031). Adjusted percentages of most health-related variables presented statistically significant differences: the LGB group reported chronic conditions more frequently (p = 0.018), higher consumption of tobacco (76.0% vs 48.2%; *p*<0.001), and psychoactive substances (52.3% vs 18.8%; *p*<0.001).

**Table 2:** Unweighted frequencies and weighted percentages of socio-demographic characteristics, chronic conditions and health-related behaviors of LGB individuals and heterosexual counterparts in the 2011 Barcelona Health Interview Survey.

	*Unadjusted %*			*Adjusted % by gender and age*		
	Heterosexual (n = 3200)	LGB (n = 77)	*p-value*	Heterosexual	LGB	*p-value*
SOCIODEMOGRAPHIC	n (%)	n (%)		%	%	
**Gender**						
Men	1528 (47.0%)	31 (42.1%)	0.401			
Women	1672 (53.0%)	46 (57.9%)				
**Age group**						
*15–34 years*	886 (28.4%)	43 (56.6%)	<0.001**			
*35–64 years*	1571 (48.7%)	26 (30.3%)				
*65 years and over*	743 (22.9%)	8 (13.2%)				
**Education level**						
*Primary or less*	1328 (42.5%)	18 (26.3%)	0.013*	29.1%	17.7%	*0*.*052*
*Secondary*	812 (25.5%)	21 (28.9%)		32.7%	31.5%	
*University or more*	1042 (32.0%)	38 (44.7%)		38.3%	50.8%	
**Social class**						
*Non manual*	1666 (56.1%)	42 (57.4%)	0.833	53.5%	55.9%	*0*.*696*
*Manual*	1297 (43.9%)	28 (42.6%)		46.5%	44.1%	
**Married or in sentimental partnership**						
Yes	1881 (58.0%)	23 (25.0%)	<0.001**	31.3%	15.8%	*0*.001**
No	1319 (42.0%)	54 (75.0%)		68.7%	84.2%	
**Country of birth**						
High income countries	2760 (87.3%)	60 (78.9%)	0.031*	82.2%	75.4%	*0*.*163*
Low income countries	428 (12.7%)	17 (21.1%)		17.8%	24.6%	
**Social support**						
*Social support (>P15)*	2690 (84.6%)	62 (77.6%)	0.095	92.0%	87.4%	*0*.*103*
*Low social support (≤P15)*	510 (15.4%)	15 (22.4%)		8.0%	12.6%	
**NUMBER OF CHRONIC CONDITIONS**						
None	1297 (42.0%)	34 (42.1%)	*0*.*187*	68.9%	63.7%	*0*.018*
One	639 (19.4%)	9 (11.8%)		19.5%	13.0%	
Two	401 (12.0%)	13 (18.4%)		7.7%	14.6%	
Three or four	427 (13.1%)	13 (17.1%)		3.0%	7.1%	
Five or more	436 (13.6%)	8 (10.5%)		0.9%	1.6%	
**HEALTH-RELATED BEHAVIORS**						
**Body mass index (BMI)**						
*Low weight or normal weight*	2103 (65.8%)	60 (75.0%)	*0*.*093*	86.8%	89.6%	*0*.*344*
Overweight or obesity	1069 (34.2%)	17 (25.0%)		13.2%	10.4%	
**Smoking**						
*Never smoker*	1748 (57.3%)	23 (25.7%)	*<0*.001**	51.8%	24.0%	*<0*.001**
*Current or former smoker*	1380 (42.7%)	52 (74.3%)		48.2%	76.0%	
**Alcohol consumption**						
*Non-drinker*	616 (26.9%)	9 (14.5%)	*0*.012*	13.7%	7.1%	*0*.*051*
*Moderate drinker*	1731 (68.3%)	48 (74.2%)		74.2%	72.2%	
*Risk drinker*	132 (4.8%)	8 (11.3%)		12.1%	20.7%	
**Psychoactive drug consumption**						
*Yes*	710 (9.6%)	44 (43.4%)	*<0*.001**	18.8%	52.3%	*<0*.001**
*No*	2490 (90.4%)	33 (56.6%)		81.2%	47.7%	

The first three columns show the unadjusted percentages, and the last three columns the adjusted percentages by gender and age. LGB: Lesbian, gay or bisexual. **Bold**: significant p-value (*p-value<0.05; **p-value<0.01).

Table 3 presents nested Tobit models with the EQ-5D index as the dependent variable. Model 1 shows that there is no significant crude difference on EQ-5D index by sexual orientation. After adjusting by gender and age (Model 2), the difference is statistically significant (-0.052, p = 0.04). Model 3 with all the sociodemographic variables shows a very similar difference (-0.055; *p* = 0.029). Once number of chronic conditions was considered (model 4), the difference on EQ-5D index by sexual orientation is no longer significant (-0.031; *p* = 0.145). After adding health-related behaviors (model 5), this difference was -0.003 (*p* = 0.899).

**Table 3:** Censored linear regression models (Tobit model) with the EQ-5D index as the dependent variable.

	MODEL 1		MODEL 2		MODEL 3		MODEL 4		MODEL 5	
	Estimate	*p-value*	Estimate	*p-value*	Estimate	*p-value*	Estimate	*p-value*	Estimate	*p-value*
**Intercept**	0.4752	*<0*.001**	0.5956	*<0*.001**	0.5482	*<0*.001**	0.5358	*<0*.001**	**0.5114**	*<0*.001**
**Sexual orientation**										
*Heterosexual*	-		-		-	*-*	-	*-*	-	*-*
*LGB*	0.0003	*0*.*990*	-0.052	*0*.040*	-0.055	*0*.029*	-0.0311	*0*.*145*	-0.003	*0*.*899*
**Gender**										
*Men*			-	*-*	-	*-*	-	*-*	-	*-*
*Women*			-0.062	*<0*.001**	-0.058	*<0*.001**	-0.0298	*<0*.001**	-0.023	*0*.002*
**Age**										
*15–34 years old*			-	*-*	-	*-*	-	*-*	-	*-*
*35–64 years old*			-0.116	*<0*.001**	-0.12	*<0*.001**	-0.0546	*<0*.001**	-0.046	*<0*.001**
*≥ 65 years old*			-0.238	*<0*.001**	-0.22	*<0*.001**	-0.0824	*<0*.001**	-0.072	*<0*.001**
**Education level**										
*Primary or less*					-	*-*	-	*-*	-	*-*
*Secondary*					0.037	*<0*.001**	0.0243	*0*.005**	0.025	*0*.010*
*University or more*					0.053	*<0*.001**	0.0280	*0*.001**	0.026	*0*.005*
**Country of birth**										
*High income countries*					-	*-*	-	*-*	-	*-*
*Low income countries*					0.029	*0*.024*	0.0088	*0*.*423*	0.001	*0*.*928*
**Married or in sentimental partnership**										
*No*					-	*-*	-	*-*	-	*-*
*Yes*					0.016	*0*.*052*	0.0192	*0*.007**	0.014	*0*.*078*
**Number of chronic conditions**										
*None*							-	*-*	-	***-***
*One*							-0.0932	*<0*.001**	-0.074	*<0*.001**
*Two*							-0.0925	*<0*.001**	-0.072	*<0*.001**
*Three or four*							-0.1452	*<0*.001**	-0.126	*<0*.001**
*Five or more*							-0.2495	*<0*.001**	-0.223	*<0*.001**
**Smoking status**										
*Never smoker*									-	
*Current or former smoker*									0.001	*0*.*890*
**Alcohol consumption**										
*Non-drinker*									-	
*Moderate drinker*									0.013	*0*.*108*
*Risk drinker*									0.028	*0*.*153*
**Psychoactive drug consumption**										
*Yes*									-	*-*
*No*									-0.025	*0*.002**
**Log-likelihood**	-1648		-1393		-1362		-1079		-802.4	
**df**	3		6		10		14		18	
**p-value**			< 0.001		< 0.001		< 0.001		< 0.001	

Estimate: EQ-5D index difference. **Bold**: significant p-value (*p-value<0.05; **p-value<0.01)

**Fig 1:**
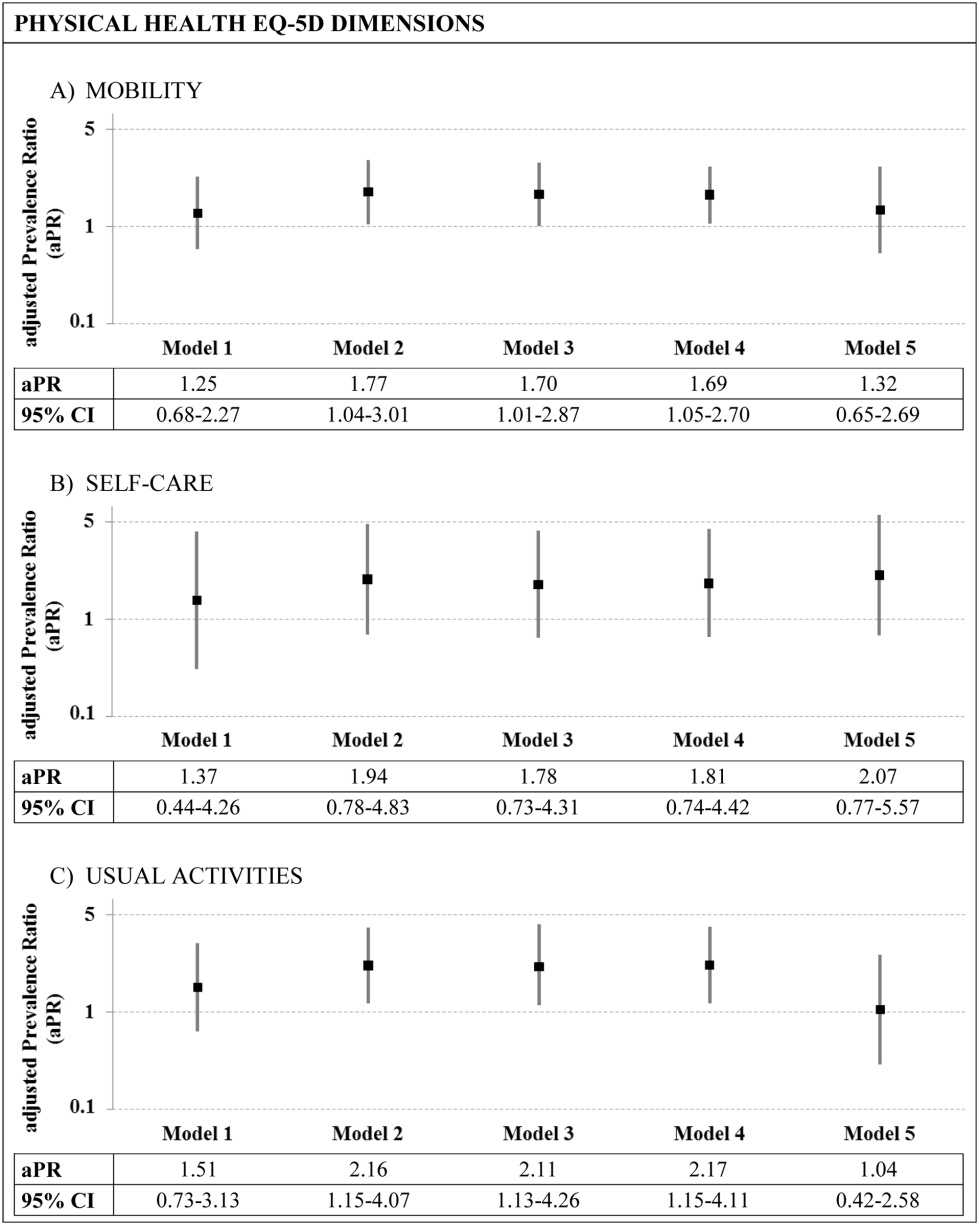
Prevalence ratios and 95% Confidence Intervals (65%CI) by sexual orientation for each physical EQ-5D dimension, considering different adjustment variables. The EQ-5D dimension (dependent variable) was dichotomized into: no problems vs moderate/extreme problems. **Model 1:** Crude prevalence ratio. **Model 2:** Adjusted by age and gender. **Model 3:** Adjusted by age and gender + sociodemographic variables (education level, country of birth, and married or in sentimental partnership). **Model 4:** Adjusted by age and gender + sociodemographic variables + number of chronic conditions. **Model 5:** Adjusted by age and gender + sociodemographic variables + number of chronic conditions + health-related behaviors (smoking status, alcohol consumption, and psychoactive drug consumption).

Nested models constructed with each of the three physical dimensions of the EQ-5D as dependent variables are shown in Fig 1. The crude prevalence ratio (Model 1) was not significant for any dimension. However, mobility and usual activities dimensions showed statistically significant differences by sexual orientation after adjusting for age and gender (Model 2), for all sociodemographic variables (Model 3), and for number of chronic conditions (Model 4). These differences were no longer statistically significant after adding health-related behaviors in model 5.

**Fig 2:**
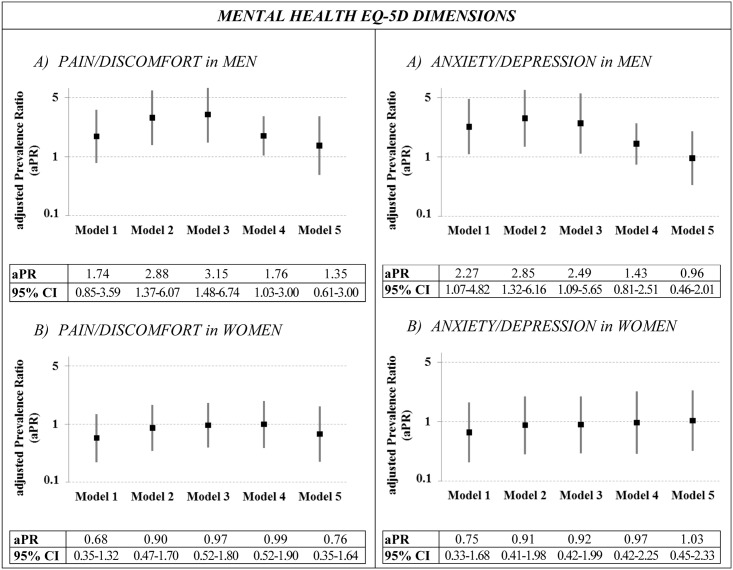
Prevalence ratios and 95% Confidence Intervals (65%CI) by sexual orientation for each mental EQ-5D dimension stratified by gender, considering different adjustment variables. The EQ-5D dimension (dependent variable) was dichotomized into: no problems vs moderate/extreme problems. **Model 1:** Crude prevalence ratio. **Model 2:** Adjusted by age and gender. **Model 3:** Adjusted by age and gender + sociodemographic variables (education level, country of birth, and married or in sentimental partnership). **Model 4:** Adjusted by age and gender + sociodemographic variables + number of chronic conditions. **Model 5:** Adjusted by age and gender + sociodemographic variables + number of chronic conditions + health-related behaviors (smoking status, alcohol consumption, and psychoactive drug consumption).

Since models of the EQ-5D’s two mental dimensions presented statistically significant interactions with gender, further models were constructed for men and women separately (Fig 2). Among men, nested models showed statistically significant adjusted prevalence ratios in models 2 and 3: for pain/discomfort aPR was 2.88 and 3.15; and for anxiety/depression aPR was 2.85 and 2.49. These differences ceased to be significant in model 5 for pain/discomfort, and in model 4 for anxiety/depression. However, no differences by sexual orientation were observed among women.

Sensitivity analysis performed after excluding the 22 participants who reported only sometimes becoming attracted to the same sex is shown in S1 File. Results of nested models with the EQ-5D index and its dimensions as dependent variables were consistent with the results obtained from the 77 individuals primarily considered in the LGB group. Also, results of the sensitivity analysis done with LGB individuals and matched heterosexual counterparts (ratio 1:5) were consistent with findings obtained through the main analysis strategy, showed in the tables and figures within the article (S2 File).

## Discussion

In our study, the LGB group clearly showed worse HRQoL than the heterosexuals. This health inequality was consistently observed in the EQ-5D index and most EQ-5D dimensions. Such pattern is common among men and women for physical health dimensions (mobility and usual activities), but differs by sex for mental health dimensions (pain/discomfort and anxiety/depression). It is important to highlight that HRQoL differences by sexual orientation disappeared when we considered chronic conditions and health-related behaviors, suggesting that these played a major mediator role.

These results support our hypotheses of worse HRQoL in the LGB population, and that the effect of sexual orientation on mental health is modified by gender. After adjusting by age, gender, and socio-demographic variables, the magnitude of the EQ-5D index difference (-0.055) is very close to the minimal important difference, estimated previously at ±0.07 for this instrument [30]. Translating this adjusted mean difference to QALYs, -0.055 is interpretable as 20 fewer days of full health per year experienced by each LGB individual. Considering the 2.3% proportion of LGB among the 1.6 million inhabitants, the total number of full health days lost each year would be higher than 700,000 in Barcelona.

Results of the previous health surveys which have explored HRQoL inequalities by sexual orientation consistently showed worse mental [3,6], but not physical [4] health for the LGB group. The United States Growing Up Today Study showed sexual orientation differences in the EQ-5D single index, without reporting mental and physical EQ-5D dimensions [9]. No SF-36 physical component differences were reported in the Dutch survey [3]. The California Quality of Life Survey [4] only showed a higher risk of poor SF-12 physical health for bisexual women and homosexually-experienced heterosexuals. In our study, in contrast to these previous findings, two of the main physical dimensions of EQ-5D, mobility and usual activities, showed consistent health inequalities in both genders by sexual orientation. The prevalence ratios indicate a 110% and 77% higher probability of having problems in usual activities and mobility, respectively, for LGB people.

Regarding mental health, the prevalence ratio for anxiety/depression in our study indicates a 185% higher probability of having problems among gay/bisexual men but not among women. Similarly, pain/discomfort dimension also presented a 188% higher probability of problems for gay/bisexual men compared to heterosexuals. Our results for men are consistent with the SF-36 mental component in the Dutch survey [3], and also with the meta-analysis of UK [6]. The latter additionally found that interaction with gender was statistically significant indicating stronger effects for men.

In our study, the LGB group was considerably younger than its heterosexual counterpart (mean 39.6 vs 48.6 years; p<0.001). Response and survival biases are two possible reasons for this age difference. First, older LGB individuals may be less likely than the younger ones to report their true sexual orientation, because during the Spanish dictatorship homosexuality was punishable with prison under the “Law of Vagrants and Crooks”. This theory is consistent with the results of our analysis comparing participants with and without information on sexual orientation, as people who did not answer the question on sexual attraction were significantly older than those who responded it. Second, there is considerable evidence of higher mortality for the LGB population [2,33], leading possibly to a survival bias. The 2001–2010 National Health and Nutrition Examination Surveys reported greater all-cause mortality for LGB than for heterosexuals (adjusted hazard ratio = 2) [33]. Another USA study [2] showing a 12-year shorter life expectancy for LGB individuals from communities with high anti-gay prejudice levels versus low ones [2] revealed suicide, homicide/violence, and cardiovascular diseases as the underlying specific mortality causes.

Results from nested models support our hypothesis regarding the effect of sexual orientation through the continuum from vulnerabilities to outcomes. Chronic conditions and risk behaviors are the principal factors explaining the HRQoL differences by sexual orientation in these models, suggesting their principal mediator role in LGB health inequalities. Statistical significance of sexual orientation disappeared after including them in the models. The Californian survey showed higher prevalence in LGB than in heterosexual participants for certain chronic conditions, especially for those related with tobacco, alcohol and drug consumption, such as asthma, heart disease, and cancer [4]. The prevalence of tobacco, alcohol and psychoactive drug consumption in our LGB subsample is around 2-fold higher than that of their heterosexual counterparts, even after adjusting by age and gender, which is consistent with evidence [5,7,17,18]. For example, in Massachusetts [18], the probability of current smoking and any 30-day drug use was also at least 2 times greater among LGB than heterosexuals; and in England [8], the LGB group reported almost 2 times higher significant alcohol and drug dependence. This pattern of worse health-related behaviors has been related to discrimination and stigma [34], which may lead to a reduction of self-control in those who feel threatened by their social identity. Minority stress theory proposes that stressors induced by homophobic culture require an individual to adapt and may affect physical and mental health [3,35], which should be considered an unfair and avoidable inequity. However, further than chronic conditions related with discrimination and substance abuse, other risk behaviors, such as unsafe sexual practices and their associated conditions should be considered as potential causes of the worse HRQoL experienced by LGB individuals.

Since data of this study came from the health survey of a large European city, results are representative of an urban setting. We have to highlight that the EQ-5D is a generic and standardized instrument which provides a simple descriptive profile and a robust single preference-based index [21,22]. In addition, the EQ-5D has proved its usefulness as a HRQoL measure for the general population [22], and detecting socioeconomic health inequalities [36]. This is the first study to have explored the mediator role of socio-demographic characteristics, health-related behaviors, and chronic conditions in health inequalities by sexual orientation.

The main limitation is that the LGB group was constructed based only on sexual attraction, without considering self-identification and sexual behavior [37]. However, sexual attraction covers some of the gaps left out by behavior or identity measures, and it has been argued that identity-based conceptualizations of sexual orientation may not adequately account for the possible variations in the population’s sexuality [37]. In addition, recent studies have shown that sexual attraction measures are more predictive than sexual identity ones to detect inequalities by sexual orientation [38]. Second, the proportion of individuals not answering the question on sexual attraction was high (7%) and part of them could be LGB. Taking into account the low number of LGB participants in the sample (2.3%), biases of non-response and social desirability affecting the sexual orientation question may have produced an infra-estimation of this group. However, the LGB percentage was similar to other developed country population surveys [3,4]. As showed previously, a sizable proportion of LGB people are unwilling to disclose their sexual orientation in surveys [39]. Consistently with our results, willingness is influenced by age, living environment (social support), education and partnership status, suggesting that general population surveys may not be fully representative of gay and bisexual populations. Third, the Barcelona Health Interview Survey design is cross-sectional, which constrains causality assessment. However, it is more plausible that sexual orientation has a negative effect on health than the inverse relationship (poor health affecting sexual orientation). Finally, the small proportion of LGB participants did not allow stratifying for age, but its interaction with sexual orientation was not significant.

## Conclusions

The LGB population presented worse HRQoL than the heterosexual one; and gender, chronic conditions, and health-related behaviors play a major role in explaining such differences. These findings support the need of including sexual orientation into the global agenda of health inequities, and provide helpful information for developing new effective public health strategies from promotion to tertiary prevention including: education based on sexual diversity, evidence-based public health interventions on general population to reduce external/social and internalized homophobia, and recommendations for health professionals to improve the LGB population’s health.

## Supporting information

S1 File Sensitivity analysis performed to test validity of sexual orientation definition by excluding participants that reported being attracted to the same sex only sometimes. (PDF)

S2 File Sensitivity analysis with LGB individuals and matched heterosexual counterparts (ratio 1:5). (PDF)
