# Supplementary material for: Health-related quality of life inequalities by sexual orientation: Results from the Barcelona Health Interview Survey
Source: PLoS One. 2018 Jan 24;13(1):e0191334. doi: 10.1371/journal.pone.0191334 (PMC5783362; doi:10.1371/journal.pone.0191334)
Supplement: S1 File — (PDF) [file pone.0191334.s001.pdf]

SUPPLEMENTARY MATERIAL 1. Sensitivity analysis performed to test validity of sexual orientation definition by excluding participants that reported being attracted to the same sex only sometimes.

Supplementary table 1. Censored linear regression models (Tobit models) with the EQ-5D index as the dependent variable. Analysis carried out after excluding participants that reported being attracted to the same sex only sometimes.

	MODEL 1		MODEL 2		MODEL 3		MODEL 4		MODEL 5	
	Estimate	p-value	Estimate	p-value	Estimate	p-value	Estimate	p-value	Estimate	p-value
**Intercept**	0.4757	<0.001**	0.5964	<0.001**	0.5494	<0.001**	0.5365	<0.001**	0.5135	<0.001**
**Sexual orientation**										
Heterosexual	-		-		-	-	-	-	-	-
LGB	-0.017	0.621	-0.061	0.037*	-0.060	0.038*	-0.0355	0.147	-0.005	0.850
**Gender**										
Male			-	-	-	-	-	-	-	-
Female			-0.062	<0.001**	-0.058	<0.001**	-0.0297	<0.001**	-0.023	0.003*
**Age**										
15-34 years old			-	-	-	-	-	-	-	-
35-64 years old			-0.117	<0.001**	-0.12	<0.001**	-0.0550	<0.001**	-0.048	<0.001**
≥ 65 years old			-0.239	<0.001**	-0.22	<0.001**	-0.0829	<0.001**	-0.074	<0.001**
**Studies level**										
Primary or less					-	-	-	-	-	-
Secondary					0.038	<0.001**	0.0248	0.004**	0.025	0.010*
University or more					0.053	<0.001**	0.0279	0.001**	0.026	0.004*
**Country of birth**										
High income countries					-	-	-	-	-	-
Low income countries					0.028	0.031*	0.0077	0.483	-0.0003	0.977
**Married or in sentimental partnership**										
No					-	-	-	-	-	-
Yes					0.016	0.060	0.0190	0.008**	0.015	0.071
**Number of chronic conditions**										
None							-	-	-	-
One							-0.0935	<0.001**	-0.074	<0.001**
Two							-0.0921	<0.001**	-0.070	<0.001**
Three or four							-0.1446	<0.001**	-0.126	<0.001**
Five or more							-0.2494	<0.001**	-0.222	<0.001**
**Smoking status**										
Never smoker									-	
Current or former smoker									0.001	0.909
**Alcohol consumption**										
Non-drinker									-	
Moderate drinker									0.013	0.124
Risk drinker									0.028	0.169
**Psicoactive drug consumption**										
Yes									-	-
No									-0.026	0.002**

Bold: significant p-value (*p-value<0.05;**p-value<0.01).

Supplementary figure 1A. Prevalence ratios and 95% Confidence Intervals (95%CI) by sexual orientation for each physical EQ-5D dimension, considering different adjustment variables. Analysis carried out after excluding participants that reported being attracted to the same sex only sometimes.

PHYSICAL HEALTH EQ-5D DIMENSIONS

A) MOBILITY

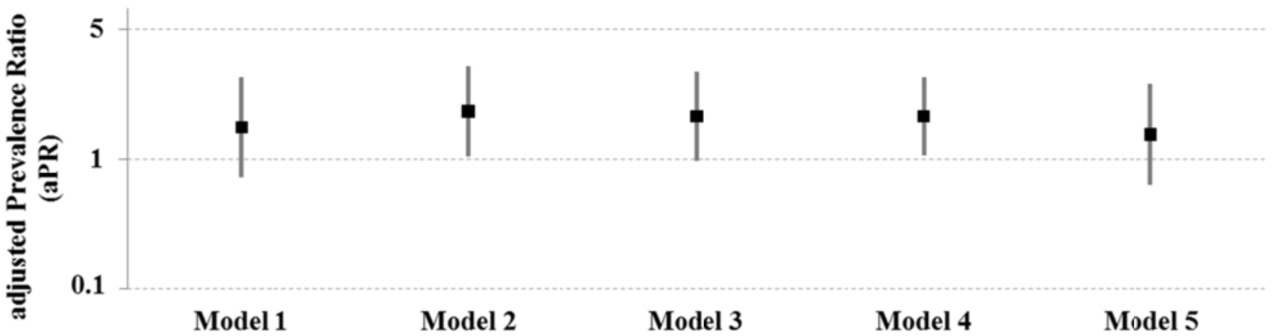

**aPR**	1.48	1.81	1.70	1.70	1.36
**95% CI**	0.79-2.77	1.04-3.15	0.98-2.94	1.05-2.77	0.72-2.55

B) SELF-CARE

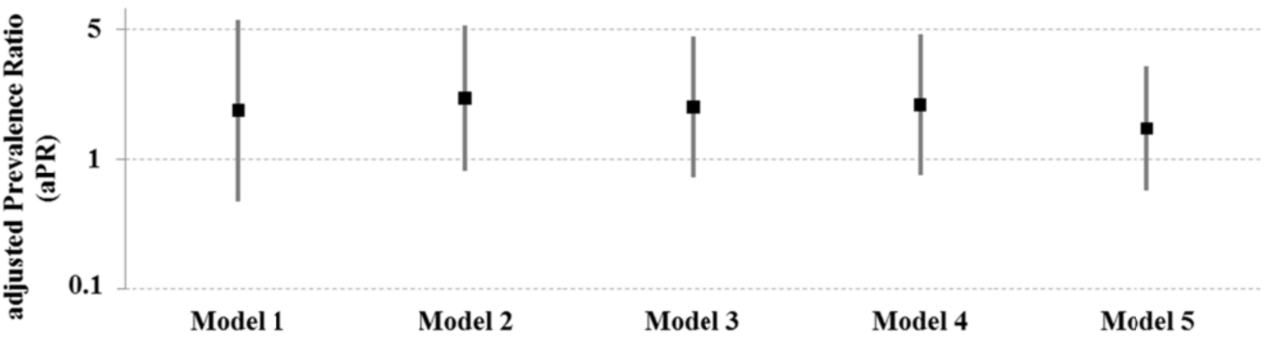

**aPR**	1.83	2.13	1.92	1.96	1.47
**95% CI**	0.60-5.62	0.87-5.22	0.80-4.57	0.82-4.68	0.68-3.16

C) USUAL ACTIVITIES

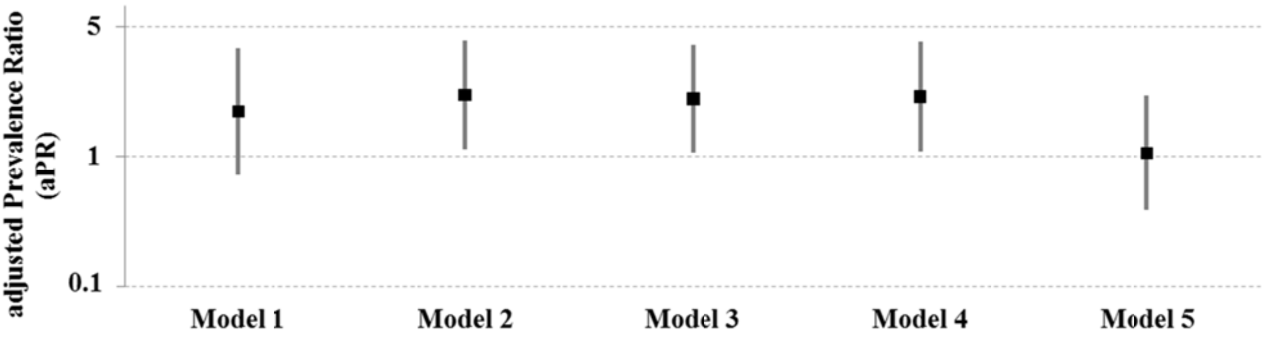

**aPR**	1.74	2.15	2.05	2.11	1.05
**95% CI**	0.80-3.80	1.09-4.23	1.05-3.99	1.06-4.17	0.51-2.13

The EQ-5D dimension (dependent variable) was dichotomized into: no problems vs moderate/extreme problems.
Model 1: Crude prevalence ratio.
Model 2: Adjusted by age and gender.
Model 3: Adjusted by age and gender + sociodemographic variables (education level, country of birth, and married or in sentimental partnership).
Model 4: Adjusted by age and gender + sociodemographic variables + number of chronic conditions.
Model 5: Adjusted by age and gender + sociodemographic variables + number of chronic conditions + health-related behaviors (smoking status, alcohol consumption, and psychoactive drug consumption).

Supplementary figure 1B. Prevalence ratios and 95% Confidence Intervals (95%CI) by sexual orientation for each mental EQ-5D dimension stratified by gender, considering different adjustment variables. Analysis carried out after excluding participants that reported being attracted to the same sex only sometimes.

MENTAL HEALTH EQ-5D DIMENSIONS

A) PAIN/DISCOMFORT in MEN

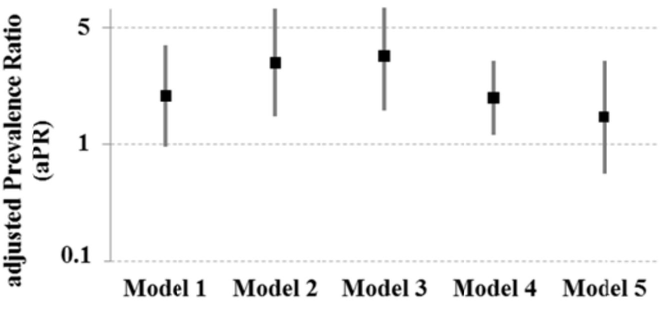

aPR	1.95	3.09	3.41	1.90	1.46
95% CI	0.96-3.95	1.47-6.49	1.60-7.28	1.13-3.20	0.67-3.18

A) ANXIETY/DEPRESSION in MEN

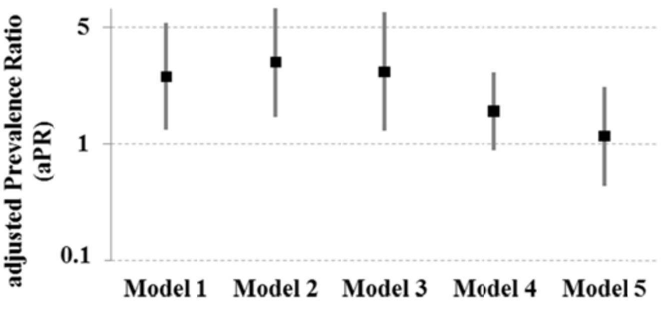

aPR	2.54	3.11	2.72	1.57	1.11
95% CI	1.22-5.30	1.45-6.68	1.20-6.20	0.91-2.71	0.56-2.19

B) PAIN/DISCOMFORT in WOMEN

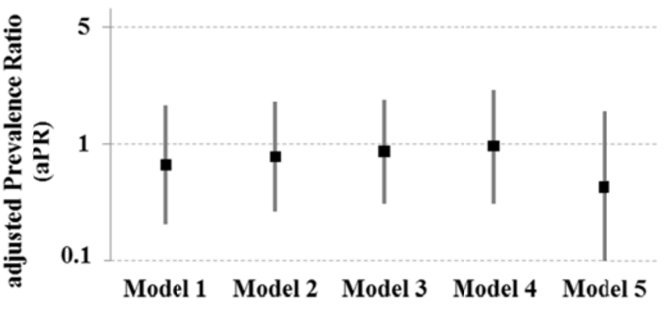

aPR	0.75	0.84	0.90	0.97	0.55
95% CI	0.33-1.71	0.39-1.79	0.44-1.86	0.44-2.13	0.19-1.58

B) ANXIETY/DEPRESSION in WOMEN

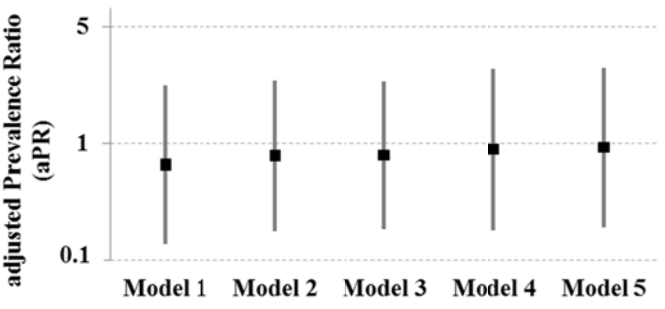

aPR	0.75	0.84	0.85	0.92	0.94
95% CI	0.25-2.25	0.30-2.38	0.31-2.35	0.30-2.83	0.31-2.84

The EQ-5D dimension (dependent variable) was dichotomized into: no problems vs moderate/extreme problems.

Model 1: Crude prevalence ratio.

Model 2: Adjusted by age and gender.

Model 3: Adjusted by age and gender + sociodemographic variables (education level, country of birth, and married or in sentimental partnership).

Model 4: Adjusted by age and gender + sociodemographic variables + number of chronic conditions.

Model 5: Adjusted by age and gender + sociodemographic variables + number of chronic conditions + health-related behaviors (smoking status, alcohol consumption, and psychoactive drug consumption).
