# Supplementary material for: Health-related quality of life inequalities by sexual orientation: Results from the Barcelona Health Interview Survey
Source: PLoS One. 2018 Jan 24;13(1):e0191334. doi: 10.1371/journal.pone.0191334 (PMC5783362; doi:10.1371/journal.pone.0191334)
Supplement: S2 File — (PDF) [file pone.0191334.s002.pdf]

**SUPPLEMENTARY MATERIAL 2. Sensitivity analysis with LGB individuals and matched heterosexual counterparts (ratio 1:5).**

**Supplementary table 2A.** Socio-demographic characteristics, chronic conditions and health-related behaviors of LGB individuals and matched heterosexual counterparts (ratio 1:5). Odds Ratios (OR) and 95% Confidence Interval (95%CI) calculated with conditional logistic regression models.

| | **LGB** | **Heterosexual** | **OR** | **95%CI** |
|---|---|---|---|---|
| | **n = 77** | **n = 385** | | |
| **SOCIODEMOGRAPHIC** | | | | |
| **Gender** | | | | |
| Men | 31 (40.3%) | 155 (40.3%) | | |
| Women | 46 (59.7%) | 230 (59.7%) | | |
| **Age group** | | | | |
| *15 - 34 years* | 43 (55.8%) | 215 (55.8%) | | |
| *35 - 64 years* | 26 (33.8%) | 130 (33.8%) | | |
| *65 years and over* | 8 (10.4%) | 40 (10.4%) | | |
| **Education level** | | | | |
| *Primary or less* | 18 (23.4%) | 98 (25.5%) | | |
| *Secondary* | 21 (27.3%) | 122 (31.7%) | 1.00 | 0.45-2.21 |
| *University or more* | 38 (49.4%) | 165 (42.9%) | 1.36 | 0.64-2.90 |
| **Social class** | | | | |
| *Nonmanual* | 42 (60.0%) | 202 (56.9%) | | |
| *Manual* | 28 (40.0%) | 153 (43.1%) | 0.95 | 0.55-1.64 |
| **Married or in sentimental partnership** | | | | |
| Yes | 23 (29.9%) | 112 (29.1%) | | |
| No | 54 (70.1%) | 273 (70.9%) | 1.19 | 0.38-3.70 |
| **Country of birth** | | | | |
| High income countries | 60 (77.9%) | 306 (79.7%) | | |
| Low income countries | 17 (22.1%) | 78 (20.3%) | 1.12 | 0.61-2.05 |
| **Social support** | | | | |
| *Social support (>P15)* | 62 (80.5%) | 328 (85.2%) | | |
| *Low social support (≤P15)* | 15 (19.5%) | 57 (14.8%) | 1.41 | 0.74-2.68 |
| **NUMBER OF CHRONIC CONDITIONS** | | | | |
| None | 34 (44.2%) | 195 (50.6%) | | |
| One | 9 (11.7%) | 81 (21.0%) | 0.66 | 0.30-1.44 |
| Two | 13 (16.9%) | 45 (11.7%) | 1.74 | 0.83-3.65 |
| Three or four | 13 (16.9%) | 44 (11.4%) | 1.96 | 0.92-4.21 |
| Five or more | 8 (10.4%) | 20 (5.2%) | **3.07** | **1.12-8.38** |
| **HEALTH-RELATED BEHAVIORS** | | | | |
| **Body mass index (BMI)** | | | | |
| *Low weight or normal weight* | 60 (77.9%) | 304 (79.2%) | | |
| Overweight or obesity | 17 (22.1%) | 80 (20.8%) | 1.09 | 0.58-2.04 |
| **Smoking** | | | | |
| *Never smoker* | 23 (30.7%) | 229 (60.4%) | | |
| *Current or former smoker* | 52 (69.3%) | 150 (39.6%) | **4.03** | **2.24-7.24** |
| **Alcohol consumption** | | | | |
| *Non-drinker* | 9 (13.8%) | 66 (22.3%) | | |
| *Moderate drinker* | 48 (73.8%) | 203 (68.6%) | 1.84 | 0.84-4.03 |
| *Risk drinker* | 8 (12.3%) | 27 (9.1%) | 2.47 | 0.82-7.43 |
| **Psychoactive drug consumption** | | | | |
| *Yes* | 44 (57.1%) | 77 (20.0%) | | |
| *No* | 33 (42.9%) | 308 (80.0%) | **5.38** | **3.17-9.13** |

Odds Ratios (OR) and 95% Confidence Interval (95%CI) calculated with conditional logistic regression models. **Bold**: significant p-value.

**Supplementary table 2B.** Censored linear regression models (Tobit models) with the EQ-5D index as the dependent variable. Analysis carried out on LGB individuals and matched heterosexual counterparts (ratio 1:5).

| | **MODEL 1** | | **MODEL 2** | | **MODEL 3** | |
|---|---|---|---|---|---|---|
| | **Estimate** | **p-value** | **Estimate** | **p-value** | **Estimate** | **p-value** |
| **Intercept** | 0.4265 | **<0.001**** | 0.4345 | **<0.001**** | 0.3735 | **<0.001**** |
| **Sexual orientation** | | | | | | |
| *Heterosexual* | - | | - | - | - | - |
| *LGB* | -0.0577 | **0.019*** | -0.0342 | 0.076 | -0.0179 | 0.406 |
| **Number of chronic conditions** | | | | | | |
| *None* | | | - | - | - | - |
| *One* | | | -0.0997 | **<0.001**** | -0.0394 | 0.122 |
| *Two* | | | -0.0875 | **0.001**** | -0.0680 | **0.014*** |
| *Three or four* | | | -0.1375 | **<0.001**** | -0.0950 | **<0.001**** |
| *Five or more* | | | -0.2228 | **<0.001**** | -0.1833 | **<0.001**** |
| **Smoking status** | | | | | | |
| *Never smoker* | | | | | - | |
| *Current or former smoker* | | | | | 0.0067 | 0.707 |
| **Alcohol consumption** | | | | | | |
| *Non-drinker* | | | | | - | |
| *Moderate drinker* | | | | | 0.0164 | 0.406 |
| *Risk drinker* | | | | | 0.0633 | 0.108 |
| **Psychoactive drug consumption** | | | | | | |
| *Yes* | | | | | - | - |
| *No* | | | | | 0.0010 | 0.959 |

**Bold**: significant p-value (*p-value<0.05;**p-value<0.01).

**Supplementary Figure 2A.** Odds ratios and 95% Confidence Intervals (95%CI) by sexual orientation calculated with conditional logistic regression models for each physical EQ-5D dimension, considering different adjustment variables. Analysis carried out on LGB individuals and matched heterosexual counterparts (ratio 1:5).

**PHYSICAL HEALTH EQ-5D DIMENSIONS**

A) MOBILITY

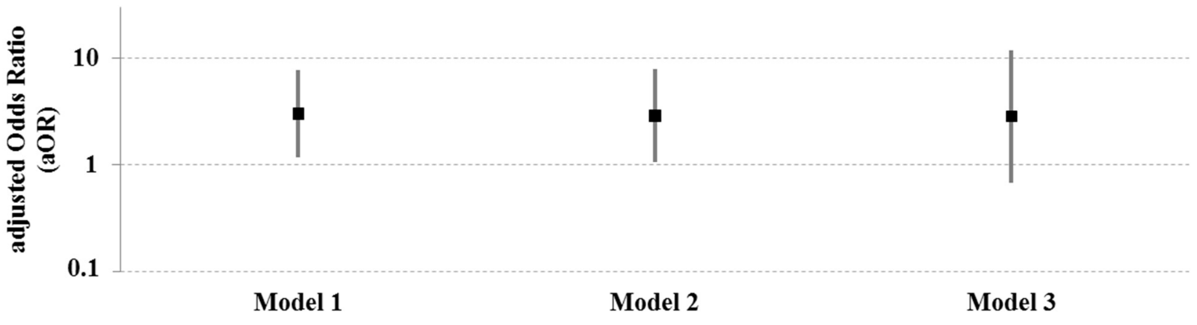


| | | | |
|---|---|---|---|
| **aOR** | 3.02 | 2.90 | 2.85 |
| **95% CI** | 1.18-7.75 | 1.07-7.86 | 0.68-11.88 |

B) SELF-CARE

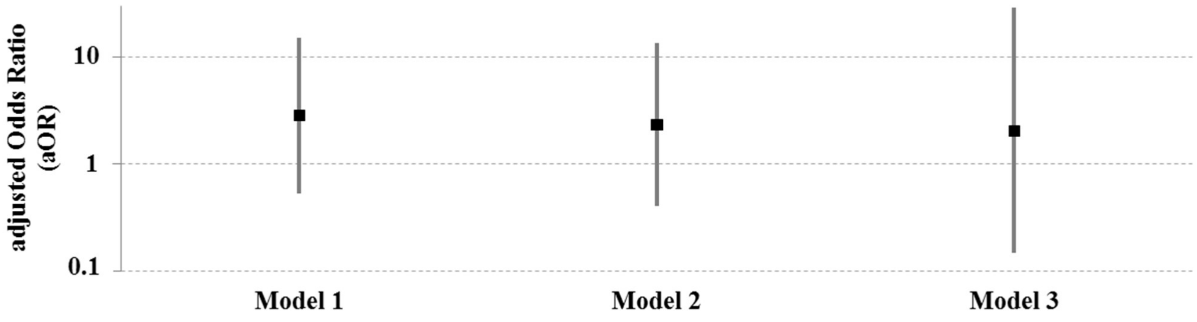


| | | | |
|---|---|---|---|
| **aOR** | 2.84 | 2.35 | 2.05 |
| **95% CI** | 0.53-15.15 | 0.41-13.46 | 0.15-28.46 |

C) USUAL ACTIVITIES

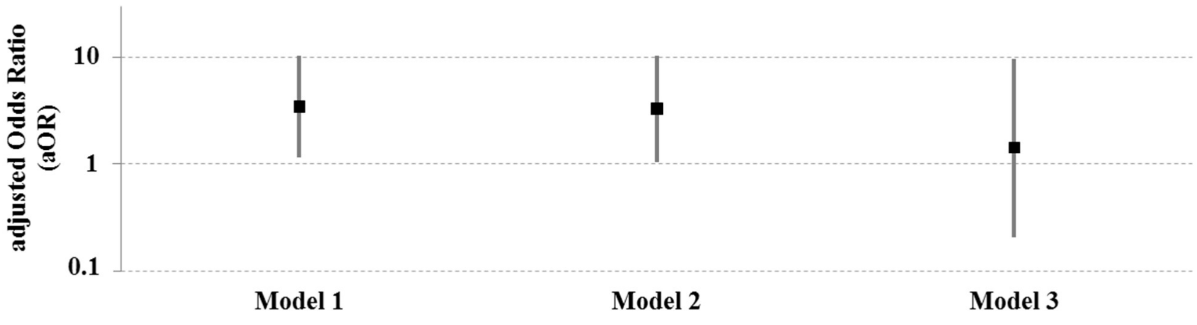


| | | | |
|---|---|---|---|
| **aOR** | 3.45 | 3.29 | 1.43 |
| **95% CI** | 1.15-10.39 | 1.04-10.41 | 0.21-9.73 |

The EQ-5D dimension (dependent variable) was dichotomized into: no problems vs moderate/extreme problems.

**Model 1:** Crude odds ratio.

**Model 2:** Adjusted by number of chronic conditions.

**Model 3:** Adjusted by number of chronic conditions + health-related behaviors (smoking status, alcohol consumption, and psychoactive drug consumption).

**Supplementary Figure 2B**. Odds ratios and 95% Confidence Intervals (95%CI) by sexual orientation calculated with conditional logistic regression models for each mental EQ-5D dimension stratified by gender, considering different adjustment variables. Analysis carried out on LGB individuals and matched heterosexual counterparts (ratio 1:5).

***MENTAL HEALTH EQ-5D DIMENSIONS***

*A) PAIN/DISCOMFORT in MEN*

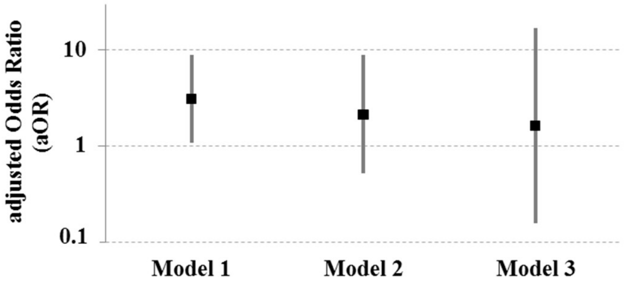


| | | | |
|---|---|---|---|
| **aOR** | 3.09 | 2.14 | 1.63 |
| **95% CI** | 1.08-8.80 | 0.52-8.83 | 0.16-17.11 |

*B) PAIN/DISCOMFORT in WOMEN*

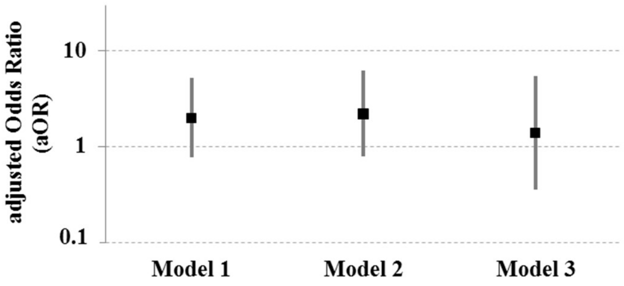


| | | | |
|---|---|---|---|
| **aOR** | 1.99 | 2.20 | 1.40 |
| **95% CI** | 0.77-5.17 | 0.78-6.21 | 0.36-5.49 |

*A) ANXIETY/DEPRESSION in MEN*

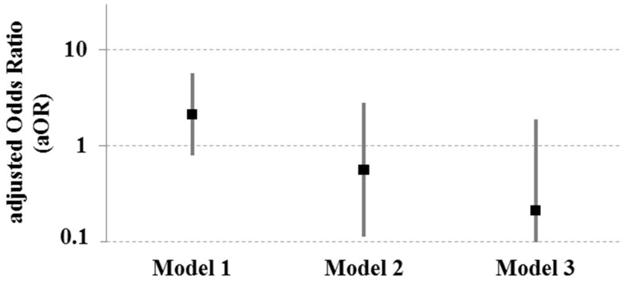


| | | | |
|---|---|---|---|
| **aOR** | 2.11 | 0.56 | 0.21 |
| **95% CI** | 0.78-5.72 | 0.11-2.82 | 0.02-1.86 |

*B) ANXIETY/DEPRESSION in WOMEN*

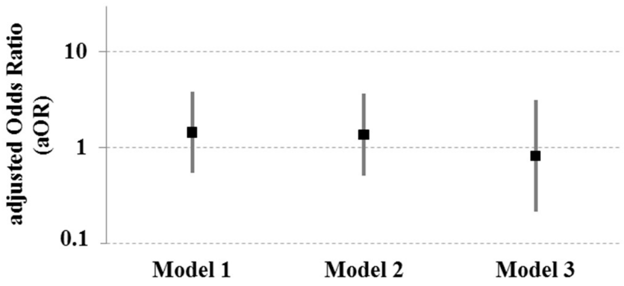


| | | | |
|---|---|---|---|
| **aOR** | 1.44 | 1.37 | 0.82 |
| **95% CI** | 0.54-3.83 | 0.51-3.69 | 0.21-3.16 |

The EQ-5D dimension (dependent variable) was dichotomized into: no problems vs moderate/extreme problems.
**Model 1:** Crude odds ratio.
**Model 2:** Adjusted by number of chronic conditions.
**Model 3:** Adjusted by number of chronic conditions + health-related behaviors (smoking status, alcohol consumption, and psychoactive drug consumption).
